# Comparing a Perceptual and an Automated Vision-Based Method for Lie Detection in Younger Children

**DOI:** 10.3389/fpsyg.2016.01936

**Published:** 2016-12-12

**Authors:** Mariana Serras Pereira, Reinier Cozijn, Eric Postma, Suleman Shahid, Marc Swerts

**Affiliations:** ^1^Tilburg Center for Cognition and Communication, Tilburg UniversityTilburg, Netherlands; ^2^Department of Computer Science, Lahore University of Management SciencesLahore, Pakistan

**Keywords:** children, eye-tracking, lie detection, methodology, motion, non-verbal signals, video analysis

## Abstract

The present study investigates how easily it can be detected whether a child is being truthful or not in a game situation, and it explores the cue validity of bodily movements for such type of classification. To achieve this, we introduce an innovative methodology – the combination of perception studies (in which eye-tracking technology is being used) and automated movement analysis. Film fragments from truthful and deceptive children were shown to human judges who were given the task to decide whether the recorded child was being truthful or not. Results reveal that judges are able to accurately distinguish truthful clips from lying clips in both perception studies. Even though the automated movement analysis for overall and specific body regions did not yield significant results between the experimental conditions, we did find a positive correlation between the amount of movement in a child and the perception of lies, i.e., the more movement the children exhibited during a clip, the higher the chance that the clip was perceived as a lie. The eye-tracking study revealed that, even when there is movement happening in different body regions, judges tend to focus their attention mainly on the face region. This is the first study that compares a perceptual and an automated method for the detection of deceptive behavior in children whose data have been elicited through an ecologically valid paradigm.

## Introduction

A question which has intrigued many generations of researchers is whether and how one is able to detect if the conversation partner is being truthful about the things he or she is claiming, or not. Apart from criminal and juridical reasons, this has been deemed relevant for educational and developmental purposes as well. In particular, there has been a specific interest in children’s deceptive behavior, as it is considered to be an important milestone in a person’s development. Typically, developing children at one point in their life “have to” learn to be able to lie, and this ability seems to emerge at similar ages, and to be ubiquitous across cultures (Talwar and Crossman, 2011).

These aspects of lying led to a series of studies into child- specific aspects of deceptive behavior (Talwar and Lee, 2002a,b; Talwar and Crossman, 2011; Fu et al., 2012; Ruffman et al., 2012), such as: the development of lies in children (Talwar and Crossman, 2011), (Talwar and Lee, 2002a), the types of lies that young children are able to tell after a transgression (Fu et al., 2012), (Talwar and Lee, 2002a), the age difference in terms of deceptive behavior (Ruffman et al., 2012), and lie detection in children (Talwar et al., 2009).

Obviously, one could think of many situations in which parents, caregivers, or teachers would find it useful to know whether or not a specific child is trying to deceive them, even when these may mostly relate to innocent issues like a broken window, a stolen cookie or a fight with another child. Yet, lie detection in children has been shown to be very difficult (Talwar and Lee, 2002a,b). There has been a specific interest in non-verbal features (such as specific facial expressions or eye-gaze patterns) that children could possibly display when they are telling a lie. However, as we will show below, in a review of the literature, the evidence regarding the usefulness of such non-verbal features as markers of deceptive behavior is quite inconclusive. The variability in reported results could partly be due to (1) the kinds of features that have been investigated in terms of their cue value and (2) the techniques that have been used to detect such features. Moreover, it would also seem important that the lies that are investigated are natural and spontaneous, and in that way representative of the behavior children exhibit in their normal social contexts, which would render acted versions of lies less suitable for research purposes.

To introduce our own approach to detecting non-verbal cues in children’s expressions, we first describe previous studies into deceptive behavior of children, then review previous findings of non-verbal correlates of lying behavior, and then say a few words about methods to (automatically) detect lies. We then embark on a description of our own study, which consists of a specific elicitation paradigm, two perception studies, and a variety of detection methods.

## Related Work

### Children’s Lying Behavior

Previous research suggests that children between 3 and 7 years old are quite good manipulators of their non-verbal behavior when lying, which makes the discrimination between truth-tellers and lie-tellers very difficult to accomplish (Lewis et al., 1989; Talwar and Lee, 2002a; Talwar et al., 2007). Most studies report that the detection of children’s lies is around or slightly above chance level, comparable to what has been claimed for adults (Bond and Depaulo, 2006; Edelstein et al., 2006).

Yet, the extent to which children display non-verbal cues could be related to the kind of lie and to the circumstances under which these are told. There is evidence that children start lying from a very young age as early as 2 1/2 years old, and lie- tellers between 3 and 7 years old are almost indistinguishable from truth-tellers (Newton et al., 2000; Talwar and Lee, 2002a). Around 3 years old, children are already able to tell “white lies”, before that they mainly lie for self-serving purposes, such as: to avoid punishment, or to win a prize (Talwar and Lee, 2002b). Nevertheless, some research suggests that lie-tellers tend to exhibit slightly more positive non-verbal behaviors, such as smiles, relaxed and confident facial expressions, and a positive tone of voice (Lewis et al., 1989). However, other research suggests that children have poor control of their non-verbal behavior, which points toward opposite and conflictive directions of what has been previously reported (Vrij et al., 2004; McCarthy and Lee, 2009). For instance, a study has reported that children between the ages of 7–9 years old show less eye contact when lying rather than when answering the truth while older children show longer eye contact, which is similar to what adults exhibit during a lying situation (McCarthy and Lee, 2009). Another study suggests a decrease of movement during a lie-tell, particularly on the hands and fingers (Vrij et al., 2004).

Furthermore, it has been reported that children tend to leak more cues to deception when they are more aware of their deceptive attempt: For example, children’s second attempts to lie (after having been told to repeat a previous lie) reveal more non-verbal cues in their facial expressions when compared to their first attempts (Swerts, 2012; Swerts et al., 2013). These findings, according to the authors, might be explained by the ironic effect of lying which states that lying becomes more difficult and most likely less successful, if a person becomes more conscious about his or her behavior when trying to intentionally produce a deceiving message.

### Non-verbal Cues to Lying

Because people are often highly skilled deceivers, accurate lie detection is in general very difficult for human judges. This means that lie detection accuracy is usually around or slightly above chance level (Bond and Depaulo, 2006; Porter and Ten Brinke, 2008; ten Brinke et al., 2012; Serras Pereira et al., 2014). However, most researchers in this field share the idea that there are certain verbal and non-verbal cues that may uncover whether a person is lying or not, and that the accuracy levels of deception detection are higher if both non-verbal and verbal cues are taken into account (Vrij et al., 2004). One line of research has been focusing on finding these cues by manipulating levels of cognitive load during a lie-tell, which makes lying more difficult, and probably facilitates the emergence of deception cues (Vrij et al., 2006, 2008). Other studies have been focusing on specific non-verbal cues of deception, which can disclose some signals related to deception, such as stress and anxiety (DePaulo, 1988; Bond, 2012). In addition, one can sometimes distinguish truth-tellers from liars on the basis of particular micro-expressions, such as minor cues in the mouth or eye region (Ekman, 2009; Swerts, 2012), like pressed lips, and certain types and frequencies of smiles (DePaulo et al., 2003). However, by their specific nature, such micro-expressions are so subtle, and last only a few milliseconds that they might escape a person’s attention, so that deception detection tends to be a very difficult task. Another study suggests that emotional leakage is stronger in masked high-intensity expressions rather than in low-intensity ones, in both upper and lower face (Porter et al., 2012). Furthermore, the highest emotional leak occurs during fear, whereas happiness shows the smallest emotional leakage. Despite the effort on finding deception cues on the face, results from many studies are frequently discrepant, and the supposed cues are often very subtle in nature (Feldman et al., 1979).

Additionally, it has been argued that eye gaze can also be a cue for deception, although the results from different studies are contradictory (Mann et al., 2002, 2004, 2013). According to one study, liars showed more eye contact deliberately than truth-tellers, whereas gaze aversion did not differ between truth-tellers and lie-tellers (Mann et al., 2013). In another study deception seems to be correlated with a decrease in blink rate, which appears to be associated with an increase of the cognitive load (Mann et al., 2002). However, in a different study, the opposite result has been reported, emphasizing that blink rate rises while masking a genuine emotion in a deceptive expression (Porter and Ten Brinke, 2008).

Body movement has also been suggested as a source for lie detection but there are some contradictory statements about the usefulness of this feature. On the one hand, some literature states that when lying, people tend to constrain their movements, even though it is unclear whether these restrictions are related to strategic overcompensations (DePaulo, 1988), or to avoid deception leakage cues (Burgoon, 2005). In a similar vein, another study measured the continuous body movement of people in spontaneous lying situations, and found that those who decided to lie showed significantly reduced bodily movement (Eapen et al., 2010). On the other hand, a study based on a dynamical systems perspective, has suggested the existence of continuous fluctuations of movement in the upper face, and moderately in the arms during a deceptive circumstance, which can be discriminated by dynamical properties of less stability, but larger complexity (Duran et al., 2013). Although, these distinctions are presented in the upper face, this study failed to find a significant difference in the total amount of movement between a deceptive and truthful condition. Moreover, when considering hand movements, another study found that lie-tellers have the tendency to do more speech prompting gestures, while truth-tellers do more rhythmic pulsing gestures (Hillman et al., 2012).

In sum, despite the fact that significant research about non-verbal cues for lie detection has been performed in the last years, results still seem to be very inconsistent and discrepant.

### Automated Methods for Deception Detection

In the past few years, several efforts have been made to develop efficient methods for deception detection. Even though there is no clear consensus on the importance of non-verbal cues (see previous section), there has been a specific interest in human face as the main source of cues for deception detection (Ekman, 2009; ten Brinke et al., 2012; Swerts et al., 2013). Many of these methods are based on the Facial Action Code System (FACS) (Ekman and Friesen, 1976), usually taken as the reference method for detecting facial movement and expressions, which has thus also been applied for detecting facial cues to deception (ten Brinke et al., 2012). As a manual method, FACS is time consuming and rather complex to apply since it demands trained coders.

More recently, automated measures are being used to help researchers to understand and detect lies more efficiently and rapidly. An example, is the Computer Expression Recognition Toolbox (CERT) which is a software tool that detects the facial expressions in real-time (Littlewort et al., 2011), and it is based on the Facial Action Coding System (FACS) (Ekman and Friesen, 1976). It is able to identify the intensity of 19 different actions units, as well as 6 basic emotions. This automated procedure to detect facial movements and microexpressions can facilitate the research of non-verbal correlates of deception, but that obviously also depends on the accuracy with which these expressions can be detected and classified. One issue is that is not immediately clear how well they would work on children’s faces.

Additionally, more novel automated measures are being used to investigate deception from different angles. Automated movement analysis is starting to be used for this purpose (Eapen et al., 2010; Duran et al., 2013; Serras Pereira et al., 2014). Eye tracking has also been used in several different ways for deception detection. Some studies (Wang et al., 2010) use eye tracking to try to define gaze patterns of liars versus truth-tellers; another option for using eye tracking systems is to study the eye-gaze patterns from the experts of deception detection. For instance, a study (Bond, 2008) has reported that experts on deception detection, when deciding about a message veracity, are perceptually faster and more highly accurate, and seem to fixate their gaze behavior in areas such as face and/or body (arms torso and legs). Likewise, some other studies have been focusing on whether deception detection can be achieved by measuring physiological data, such as brain activity, galvanic skin conductance, and thermography techniques (Kozel et al., 2005; Ding et al., 2013; Van’t Veer et al., 2014). However, these methods are quite intrusive, and not suitable for all contexts, especially when dealing with specific types of population, such as children.

### Current Study

In sum, considerable work is currently being done on the development of efficient automated methods to detect deception, but there is still a tendency to discard the body as a source of possible non-verbal cues. In the future, such methods could be combined with what has been achieved via automated analysis of verbal cues (Benus et al., 2006) and gestures (Hillman et al., 2012) as potential sources for lie detection, since combining verbal and non-verbal cues have proven to be more accurate for lie detection (Vrij et al., 2004). Moreover, the inconsistency regarding the relevance and value of bodily cues for deception may partly be due to the use of different detection methods. This discrepancy is worthy to be investigated in a more systematic approach.

Finally, most of the research with children focuses on developmental questions of lying. In this study, we are interested in exploring the non-verbal cues of such behavior based on the assumption that children are less formatted by the social rules, and that they tend to leak more cues to deception when they are more aware of their deceptive effort (Swerts, 2012). Based on what is above described, this study presents a new approach to look into non-verbal cues of deception. It investigates how easily it can be detected whether a child is being truthful or not in a game situation, in which the lies are more spontaneous, and much closer to a normal social context. In addition, it explores the cue validity of bodily movements for such type of classification, by using an original methodology – the combination of perception studies and automated movement analysis.

## Methods

### Paradigm for Eliciting Lies

In order to elicit deception in young participants, we used a child-friendly procedure, which naturally induces truthful and deceptive statements from children. Inspired by previous work (Talwar and Lee, 2002a,b; Talwar and Crossman, 2011), we developed a specific game, “Guess what I have behind the back?” which was presented to a child participant as a game in which an adult person (experimenter) had to guess what kind of object (fruit or animal) the child participant was hiding behind his/her back. This was achieved by a series of nine simple questions (is it a fruit or an animal? What is its color, etc.) asked by the adult, and answered by the child. After the series of questions, the experimenter had to make a guess about what object the child was hiding. In the truthful condition, the child that hid the object replied to the questions about the object in a truthful way (truthful condition). In the two subsequent lying conditions, the child was encouraged to lie (by giving incorrect answers about the object, such as: saying that the object was orange when it was red) when answering the questions about the object. In order to achieve this, a confederate (another adult who was also present in the room) in between sessions prompted the child to lie in order to win the game and get a present as a reward. The arguments given by the confederate to elicit the lie were that the experimenter thought and said out loud that she was the best in this game. The confederate did this when the experimenter was absent, because she had to leave the room with an excuse (to pick up a phone call, or to pick up the next child that would play the game). The game was played twice in the deceptive condition, the only difference being that during the first lying condition the experimenter lost the game (after the final question) and guessed the object wrongly; while in the second lying condition, despite what the child described, the experimenter guessed the object correctly. The reason for having two lying conditions was inspired by previous results that children’s second attempts of deceiving might reveal more non-verbal cues (Swerts et al., 2013). Each object (banana, apple, dog, and a giraffe) was attributed to a specific box, so that the experimenter always knew what was inside the box (even when the child was not aware that the experimenter in fact had this knowledge).

#### Participants

Forty-two Portuguese children aged between 6 and 7 (*M* = 6.38) years old enrolled in the 1st year of primary school participated. Two of the participants (a boy and girl) were removed from the sample because they refused to deceive the experimenter.

#### Procedure

Each game session lasted for about 30 min (depending on how wordy or fast a specific child was), and consisted of five distinctive moments: (1) Briefing, (2) Warming-up; (3) Truthful condition (Tc), (4) Lying conditions (Ly1 and Ly2), and (5) Debriefing. In the first phase (briefing), the experimenter explained the game to the children. In the warming-up, the experimenter played the game with the child, but in this case the roles were inverted: the experimenter picked an object and hid it behind her back. Then, the child had to ask questions about the object until the child was able to guess what the object was. After this training session, the actual experiment started (phases 3 and 4). First, the child played in the truthful condition, and then in the two lying conditions (see above). The session ended with a short debriefing in which a small reward was given. All the children enjoyed the game, and engaged easily (without any suspicion) on the lies.

#### Recordings

**FIGURE 1:**
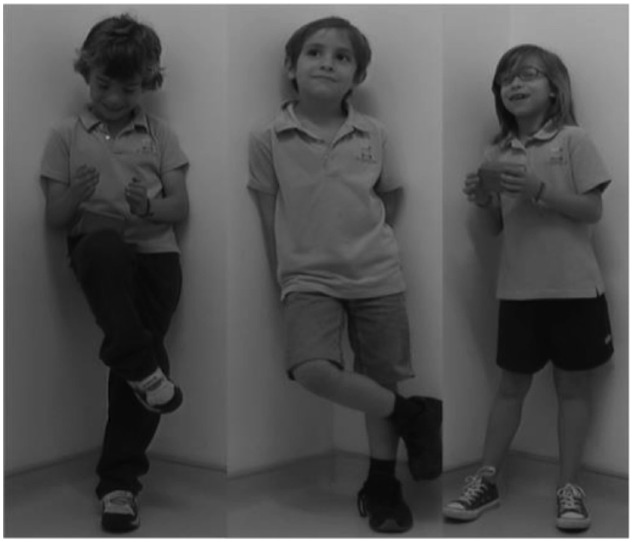
**The figure displays three different children playing the game during the experiment**.

The games were recorded in high definition (HD) color using an HD video camera. Only the child was recorded (frontal view), while the experimenter, who was positioned next to the camera, was not recorded. Children were standing upright (**Figure 1**), against a white wall, to assure that all body movements were captured during the game play. The sessions with the children lasted between 52 s and 2.30 min.

#### Ethical Consideration

At the time of the data collection, there was no formal ethical approval from the university department, since only recently an ethical committee was set up. Nevertheless, a complete and rigorous process was respected and followed up during the realization of the experiments. First of all, we got approval form the school pedagogical director, and after this parents were also informed about the goal of the experiment. Secondly and prior to the experiments, signed consent forms from the children’s parents were collected, in which it was asked permission for each child to participate and to be recorded. It also stated that the data and recordings of the children would be treated with confidentiality, and that would only be used for scientific purposes, such as articles and conferences presentations. In the end of the experiments, we also debriefed children and teachers (school staff).

### Perception Test

A perception test was set up in order to explore whether judges would be able to guess whether the recorded children were saying the truth or were lying to the experimenter, based on their non-verbal behavior. From the 40 children, fragments of 30 children were selected for the perception test. For each child, we selected its responses to two consecutive questions (“is it a fruit or an animal?” and “what is the size of it?”) in the three elicitation conditions, leading to a total of 90 clips. These two consecutive questions were chosen to have a balance between an open and closed question, and because using all nine questions would create extremely long stimuli for subjects, which would cause tiredness and distraction effects during the task performance. In addition, ten children were not included in the perception test because they took more than 20 s in replying to the above-mentioned questions, so that their responses became atypically long. Finally, the clips (without sound) were presented in a randomized order to small groups, consisting of 2–3 participants. The audio was removed, as we were primarily interested in the non-verbal expressions, and wanted to make sure that people could not rely on lexico-syntactic cues when making their judgments. In addition, the judges were not informed about the relative frequency of truthful and deceptive utterances.

#### Participants

Twenty undergraduate students, between 18 and 25 years old (*M* = 22.2, 15 women), were recruited from the online subject pool system from the School of Humanities of Tilburg University. Students participated for course credit.

#### Procedure

Upon arrival in the lab, each participant was informed about the aim of the perception test. Every participant also received a questionnaire for rating each clip. The questionnaire consisted of two simple questions: (1) Is this child lying? (yes/no); and (2) If you said “yes,” where did you base your decision on? (feet/legs/shoulders/face/other, please specify). When responding to the second question, multiple answers were allowed. The perception test was administered as a Keynote presentation on an iMac. The perception test consisted of two phases – the warming-up phase in which three test clips (different from the ones used in the actual experiment) were shown and the respective part of the questionnaire was completed. After this the actual perception test started, in which 90 clips were presented and the respective questionnaire had to be completed. After each clip, there was a response interval of 12 s, which participants used to rate the clip. Each session was group-paced, though each participant had to do the task individually, and lasted between 35 min.

#### Results

The following results refer to the first question of the questionnaire – Is this child lying? (yes/no). For each clip, we first computed the percentage of times it had been classified as being deceptive by the judges. In an ideal situation with perfect classification results, this would give a response of 0 for clips of the truthful condition, and 100 for the two lying conditions. A one-sample *t*-test on these average scores revealed that they differed significantly from chance level (50%). In particular, the test showed that the scores were significantly below 50% for the truthful condition [*t*(19) = -2.27, *p* = 0.05], and above 50% for the two lying conditions [for the Ly1, *t*(19) = 5.01, *p* = 0.05; and for Ly2 *t*(19) = 3.91, *p* = 0.05].

**FIGURE 2:**
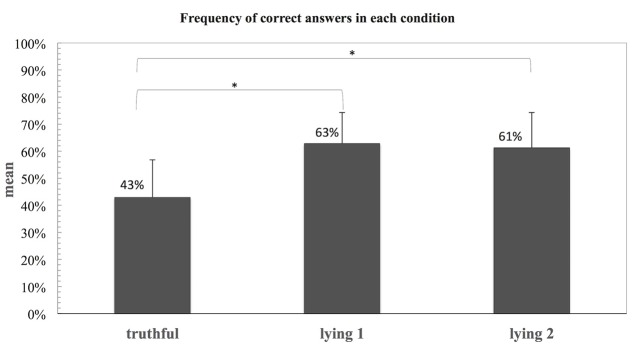
**Frequency of lie responses for each of the three conditions (Tc, Ly1, and Ly2) in experiment 1.** Statistical significant difference, ^∗^*p* = 0.001.

In addition, a Repeated Measures Anova was conducted to compare the percentages of lie responses in each of the three conditions (Tc, Ly1, and Ly2). The analysis revealed a main effect of condition [*F*(2,38) = 38.80, *p* = 0.001]. *Post hoc* pairwise comparisons using the Bonferroni method showed that Ly1 (*M* = 0.63, *SD* = 0.11) and Ly2 (*M* = 0.61, *SD* = 0.13) were significantly different from the Tc (*M* = 0.43, *SD* = 0.14), but not between themselves (Ly1 vs. Ly2). These results are depicted in **Figure 2**.

The goal of the second question- If you said “yes,” where did you base your decision on? (feet/legs/shoulders/face/other, please specify), was to understand which part(s) of the body judges thought to be meaningful for deciding whether a child is lying or not. The relative frequency for each of the reported areas of the body was calculated for all the lying clips and perceived lies (the ones that actually were truthful but were reported by the judge as a lie). Results showed that participants reported that the face (75.62%) is the best assumed indicator of a lie, but feet (33.40%) and legs (30.35%) also were thought to be meaningful, while shoulders (16.63%) and other (12.71%) seemed to have less significant impact. Note that these observations were based on an overall analysis of the child data, even though it was clear that there were idiosyncratic differences between the participants (e.g., with some children being more expressive than others).

### Automated Movement Analysis

**FIGURE 3:**
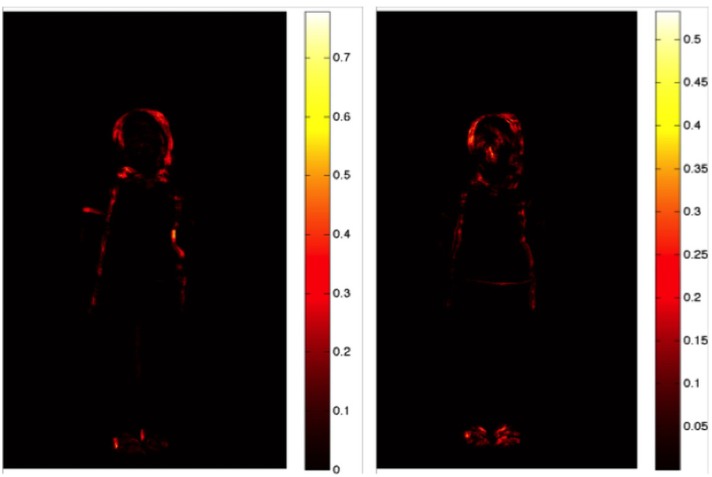
**Illustration of the heat maps showing the outline of the body of a girl obtained for a truthful **(Left)** and a deceptive **(Right)** sequence in experiment 1.** The unit of measure is the average pixel change, meaning that brighter colors indicate larger changes during the video, i.e., more movement.

In order to estimate the amount of movement in the video sequences and to identify which areas of the body show those non-verbal cues, a frame-differencing method was used. In this automated method, the absolute changes of (gray-level) pixel values in all pairs of subsequent frames are recorded and averaged per pixel over the entire video sequence yielding for each video a heat map showing the averaged changes during the sequence (see **Figure 3**). See Supplementary Material for the script used for this method. A heat map is a visual representation in which numerical values, in this context average pixel changes, are represented by colors that are easily associated with an increasing quantity. In the present case, the colors reflect increasing temperatures ranging from black/brown (low), via yellow (intermediate) to white (high).

The video dataset used in the perception test was submitted to an automated computer analysis. In total there were 30 participants, resulting in 3 × 30 videos matrix. Each triplet consists of one video per condition: truthful (Tc), first lying (Ly1), and second lying (Ly2). The videos were cropped in order to retain the central region showing the interviewed child. The original size of 1920 pixels × 1080 pixels was reduced to the central region of 801 pixels × 1080 pixels. In three cases, small additional portions were removed due to movements caused by the experimenter and assistant.

In addition, to suppress spurious motions due to illumination compensation in the video camera, pixel changes were threshold. The threshold value was set at a fixed value of 25 (absolute pixel-change range: 0–255). All change values smaller than the threshold were set to zero. A visual assessment of all heat maps revealed that this thresholding effectively removed the spurious motions for all videos, while retaining the child-induced motions.

The estimated total movement is expressed in the absolute pixel change, which is obtained by taking the average of the average pixel change maps. **Figure 3** displays two heat maps of the average pixel changes obtained for a truthful (left) and a deceptive sequence (right). The first image (left side) is a truthful sequence whereas the right side corresponds to a lying sequence. For the truthful condition, it is possible to observe that the movements occur mainly on the upper part of the body and the head, while the heatmap for the deceptive condition shows that the movements mainly occur on the head, face and feet. The brighter feet are due to their frequent movements during the video sequence.

#### Results

To assess the relation between the percentages of lie responses of the judges (from the perception test) in each of the three conditions and the amount of movement estimated by the frame-differencing method, a Spearman correlation analysis was performed. According to this analysis, there was a statistically significant correlation (*r*_s_ = 0.46, *n* = 90, *p* < 0.001) between these variables suggesting that the more movement there is in a clip, the more likely it is that a clip is perceived as lie. Note that this first test did not specify whether a specific clip was in fact a lie or not, only that lie responses (whether correct or not) correlate with the movement measure.

A Wilcoxon signed rank test of the automated movement results for each condition (Tc, Ly1, and Ly2) was performed to assess whether these movement scores could distinguish each of the conditions. The comparison between the truthful and the first lying condition showed that the pairwise differences were not statistically significant (*Z* = -0.48, *p* = 0.61, *r* = 209). However, the results obtained by compared the truthful and second deceptive conditions showed a much clearer pattern, which suggested predominance of movement in the second deceptive condition, confirmed by the Wilcoxon signed rank test revealing the difference to be significant (*Z* = -2,56, *p* = 0.01, *r* = 108).

Additionally, a body-region based analysis was performed to further understand whether the movement analysis would reveal differences in performance for the different body parts. Three regions, namely head, trunk, and legs were individually analyzed by (i) manually defining the horizontal boundaries (by taking the average frames of each video and interactively setting the horizontal boundaries by means of an interactive script) in each heat map that separate head from trunk and trunk from legs, and (ii) computing for each of the three regions the mean average pixel change value as a measure of amount of movement. A Spearman correlation analysis was conducted in order to evaluate the relation between the percentages of lies responses from the judges in each of the three conditions and the amount of movement per region (head, trunk, and legs) calculated by this method. Results showed a statistical significance between each body region and the percentage of lies responses from the perception test (head: *r*_s_ = 0.38, *n* = 90, *p* = 0.001; trunk: *r*_s_ = 0.45, *n* = 90, *p* < 0.001; legs: *r*_s_ = 0.40 *n* = 90, *p* < 0.001), which was in line with the previous analysis, indicating that the more movement there is in each of these regions, the more probable it is that a clip is perceived as lie. Furthermore, this analysis also showed that each region had a weaker correlation when compared to the overall movement correlation (*r*_s_ = 0.46, *n* = 90, *p* < 0.001), although the trunk correlation (trunk: *r*_s_ = 0.44, *n* = 90, *p* < 0.001) was closer to the overall movement correlation.

To evaluate whether the movements scores in each of the three regions (head, trunk, and legs) could differentiate each of the three conditions (Tc, Ly1, and Ly2), a Wilcoxon signed rank test was conducted. For the three regions, the comparison between the truthful and the first lying condition showed no statistical significance on pairwise differences (head: Z = -0,11, *p* = 0.91; trunk: *Z* = -0.71, *p* = 0.48; legs: *Z* = -0.10, *p* = 0.30). On the other hand, when comparing the pairwise differences between the second lying condition and truthful condition for the three regions of the body, results showed a prevalence of movement in the second lying condition, (head: *Z* = -2.21, *p* = 0.02; trunk = *Z* = -2.40, *p* = 0.01; legs = *Z* = -2.52, *p* = 0.01).

Finally, when comparing the movement differences between different regions in each of the conditions, it was possible to observe that for each of the three conditions, there was a statistical difference between the head and legs regions (Tc: *Z* = -3.73, *p* = 0.00; Ly1: *Z* = -2.28, *p* = 0.02; Ly2: *Z* = -3.32, *p* = 0.00), and between the trunk and legs (Tc: *Z* = -4.06, *p* = 0.00; Ly1: *Z* = -3.88, *p* = 0.00; Ly2: *Z* = -4.08, *p* = 0.00], suggesting a predominance of movement on the upper part of the body; while there was not a statistical significance between the movement of the head and the trunk in each of the three conditions (Tc: *Z* = -0.71, *p* = 0.48; Ly1: *Z* = -1.02, *p* = 0.31; Ly2: *Z* = -1.53, *p* = 0.12).

## Second Study

### Eye Tracking Study

The results from the first study showed that the face is assessed (by the judges) to be the best region to detect a lie, and that there was more movement happening on the body (in all the three regions) in the second lying condition.

In order to further comprehend these outcomes, an eye tracking study was setup. The main purpose was to understand whether the judges’ gaze patterns – where they actually looked – when deciding whether one was lying or not would be in line with their own intuitions, especially in view of the fact that other body parts could in principle also be informative. And, to see if these gaze patterns were congruent with what was previously reported on the first perception test, mainly if the face is the principal region to where they looked; or if there is less conscious observation behavior while looking at different parts of the children’s body.

To achieve this, judges’ eye movements were recorded with an SMI Hi-Speed Eye-Tracker with a sample rate of 250 Hz, on a new set of participants who also did the perception task (see below).

#### Stimuli

Due to the fact that eye-tracking studies are very demanding to the eyes, the amount of clips used for this experiment was shortened. From the 30 children from the first perception study, 20 randomly children in the three elicitation conditions (Tc, Ly1, and Ly2) were selected, leading to a total of 60 clips. Finally, the clips (without sound) were presented in a randomized order to participants.

#### Participants

Twenty-seven Dutch undergraduate students, between 18 and 42 years old (*M* = 22.1, 25 women), were recruited from the online subject pool system from the School of Humanities of Tilburg University. Students participated for a half course credit. Eight students were excluded from the sample, either because they did not meet the experiment requirements, or because at a certain point of the experiment, they could not get calibration or validation accuracy lower than 1.5 degrees of freedom in both axis (*X*,*Y*).

#### Procedure

Upon arrival in the lab, each participant was informed about the aim of the test. The perception test consisted of two phases – the warming-up phase in which three test clips (different from the ones used in the actual experiment) were shown, so that the judges could get acquainted with the experiment setup. After this, the actual perception test started, in which 60 clips were presented. Subsequently, the participants had to answer (on the screen) always the question: (1) Is this child lying? (yes/no). Each session was self-paced, and lasted between 30 to 40 min, with a break of 5 min in between. The break was created as an attempt to eliminate the possible fatigue of the eyes that such system can cause. There were two 9-point calibrations, one in the beginning of the experiment, and the second after the break. There were also three validations, one after the warm-up phase, the second one in the middle of the first part, and the last one in the middle of the second part. The accepted gaze position error was below 1.5 degrees of freedom. Nevertheless, due to the length of the experiment that occasionally caused tiredness in the eyes, each attempt for calibration and/or validation was repeated maximally three times; otherwise, participants were excluded from the experiment.

### Apparatus

The perception test was administered on a Dell screen (1650 × 1050) with an SMI RED 250 eye tracker, running at 250 Hz. The experiment was setup in Experimenter Suit 360, which is a software component of SMI-Tracker.

### Data Processing

The eye gazing data was processed in BeGaze 3.5. For each clip, four subjacent areas of interest in the children’s body were drawn. These areas had to be manually defined for each clip, mainly because most children had different sizes, and were positioned in slightly different areas of the screen. These areas corresponded to the same body regions that were used for the second question from the first perception test (If you said “yes,” where did you base your decision on? feet/legs/shoulders/face). The first area contained the child’s head and neck, the second area covered the child’s upper body (from the shoulders to the hips), the third area was defined by the legs (from the hips to the ankles), and the fourth area enclosed the feet. Additionally, a fifth area on the left low corner of the screen was defined, and considered to be noise (occasionally the hands of the experimenter appeared on that area). Also, screen shots of each clip were made, preserving the same size and image quality as the original clip, so that the areas of interest could be exported on top of each screen shot, and keep the right position on the children’s body. Finally, the gaze data from the eye-tracker, the areas of interest and the print screens were exported to Fixation. Fixation is a software tool that allows for easy analysis of eye movements and data preparation for statistical testing (Cozijn, 2006).

In Fixation, a manual review of all fixations and a reassignment of some fixations into the respective areas of interest was made; the reason for this was related to the fact that the movement in the clips is not contemplated on a screen shot of the clips (which are static images of the clips). Therefore when exporting all the data into Fixation, there were some fixations that fell very close to the areas of interest, but not exactly in the areas of interest, and those required a manual correction and reassignment for the respective area of interest, if that was the case. Fixation allows such reassignments to be made.

### Results

In order to verify the lie detection accuracy of this new set of judges, and confirm if they behaved similarly to the first group of judges (first perception test), a similar analysis for the question – Is this child lying? (yes/no) was performed. Based on the percentage of times that each clip had been classified as being deceptive by the judges, a one-sample t-test showed that the scores were significantly below 50% for the truthful condition [*t*(18) = -4.11, *p* = 0.05], and above 50% for the two lying conditions [for the ly1, *t*(18) = 1.01, *p* = 0.05; and for ly2 *t*(18) = 2.56, *p* = 0.05].

**FIGURE 4:**
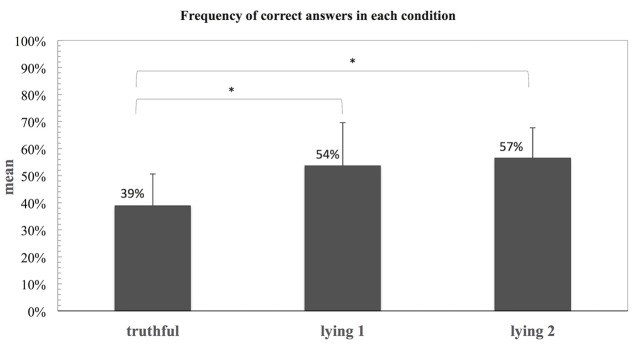
**Frequency of lie responses for each of the three conditions (Tc, Ly1, and Ly2) in experiment 2.** Statistical significant difference, ^∗^*p* < 0.001.

When comparing the percentages of lie responses in each of the conditions (Tc, Ly1, and Ly2), a Repeated Measures Anova revealed a main effect of condition [*F*(2,36) = 17.29, *p* < 0.001]. *Post hoc* tests using the Bonferroni correction revealed Ly1 (*M* = 0.54, *SD* = 0.16) and Ly2 (*M* = 0.57, *SD* = 0.11) were significantly different from the Tc [*M* = 0.39, *SD* = 0.12, but not between themselves (Ly1 vs. Ly2)]. These results are depicted in **Figure 4**.

In order to compare the gaze duration in each of the four body regions (head, trunk, legs, and feet) for the three conditions (Tc, Ly1, and Ly2), a Repeated Measures Anova was performed. **Table 1** shows the gaze duration for the four different regions (head, trunk, legs, and feet). Results revealed that judges gazed significantly more often to head region [*F*(1,19) = 96.52, *p* = 0.001] than to other body parts, but there was no interaction between the three conditions and each of the four regions, neither between each region and the quality of the observers’ rates (good vs. bad judgment regarding the rate accuracy). For the head region, *post hoc* tests using the Bonferroni correction revealed that Tc (*M* = 126.9, *SD* = 46.6), Ly1 (*M* = 106.5, *SD* = 28.4) and Ly2 (*M* = 124.9, *SD* = 28.5) were significantly different from other body regions Tc (*M* = 45.4, *SD* = 32.1), Ly1 (*M* = 34.9, *SD* = 31.1), and Ly2 (*M* = 42.1, *SD* = 30.9). In other words, even when our previous movements analyses suggested that cues to deception appear to be distributed over the whole body (head, trunk, and feet), the judges only seemed to pay attention to cues that appeared in the child’s facial area. Note, however, that the eye fixations on the head do not imply that the judges did not notice cues in other body parts, but it does suggest that the face is intuitively used as the primary resource for lie detection.

**Table 1:** The average of the gaze duration in each of the four regions in seconds.

Body regions	Tc Mean (SD)	Ly1 Mean (SD)	Ly2 Mean (SD)
Head	126.9 (46.6)	106.5 (28.4)	124.9 (28.5)
Trunk	35.6 (28.4)	27.4 (27.8)	31.3 (29.6)
Legs	3.02 (4.03)	2.15 (2.89)	4.70 (4.81)
Feet	0.76 (1.03)	0.64 (1.11)	0.41 (0.44)


**FIGURE 5:**
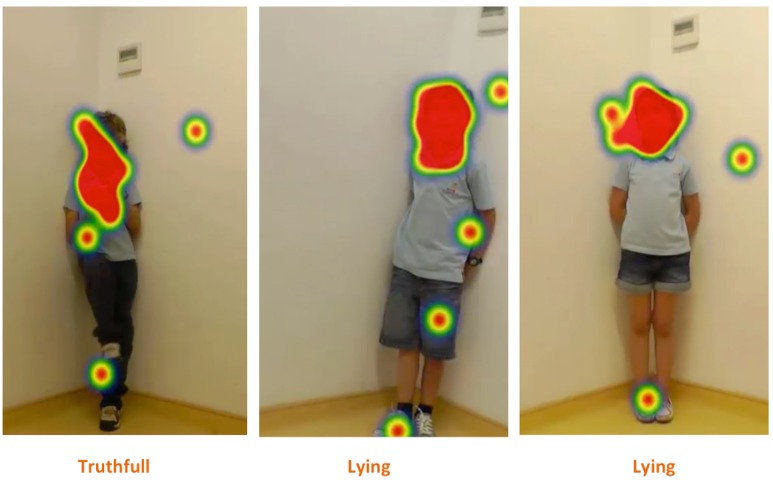
**Illustration of a heat map showing all the judges’ fixations in each of the conditions three conditions (Tc, Ly1, and Ly2)**.

**FIGURE 6:**
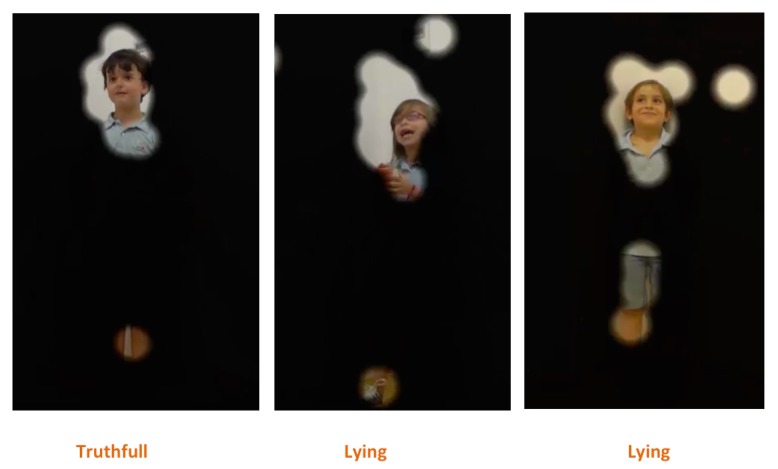
**Illustration of a focus map showing the regions (in black) that had fewer fixations by all the judges in each of the three conditions (Tc, Ly1, and Ly2)**.

The heat map in **Figure 5** represents the judges’ fixations in each of the conditions, which clearly illustrates that independent of the condition (Tc, Ly1, and Ly2), the main hot spot is on the children’s face, meaning that that was the region where judges spent the majority of the time. Likewise, there were smaller hot spots in other parts of the body, such as legs and feet, suggesting that the judges looked at those regions when there was some movement happening there, even when to a far lesser extent. Finally, a similar result is also depicted in the focus map on **Figure 6**. The focus map shows the regions that were less visualized (covered in black) by the judges, i.e., that had less fixations in each of the three conditions (Tc, Ly1, and Ly2), which also illustrates that the face was the most prominent region, but once again there were also uncovered areas in the legs and feet regions.

## Discussion of Results

Previous studies have shown that results regarding lie detection in children are very discrepant, and often (self) contradictory. These inconsistencies might not only be explained by the idiosyncrasy of lies, but also because there is such variability in the methods used to investigate it. In addition, as already pointed out, the tendency to discard the body, and the relevance of bodily cues may also contribute to these facts. As an attempt to address these issues, the present study uses a novel and systematic approach to look into non-verbal cues to deception, by combining a game elicitation paradigm for lie elicitation with an original methodology of perceptual and automated vision-based analyses.

As a basis for our study, we used behavioral data that were obtained through a game-based procedure, that worked extremely well with our participating children, since a vast majority of them spontaneously engaged in the game and were eager to lie in order to win. However, we have only looked at children who were between 6 and 7 years old, which naturally begs the question how their behavior compares to that of people in different age ranges. There is evidence in the literature that suggests that children’s lying behavior develops with age (Talwar and Crossman, 2011), probably related to their more general cognitive and moral development, but details are lacking on how exactly their lying behavior evolves toward adulthood. To the best of our knowledge there are actually no studies exploring the differences between adults and children’s lying behavior. But while it is clear that the research questions regarding such developmental patterns are interesting and relevant for the study of cognitive and moral development in general, it is not self-evident what paradigm would work in similar ways with participants in different age groups. Our current game-based elicitation procedure was tuned to younger participants, but would literally seem to “childish” to be used with adult participants, whereas other paradigms may work well with adults, but may not be child-friendly. An important experimental challenge for the future is therefore to find a method that is able to obtain comparable behavior from children and adults in truthful and deceptive contexts.

Our research has led to a number of interesting results. First, it is noteworthy to point out that in both studies, participants were able to distinguish truthful clips from lying clips above chance level, although the percentage of accuracy for lie responses was lower in the second study, which could be due to the smaller amount of clips presented to the judges (on the first study 90 clips were shown vs. 60 clips in the second study), and the fact that the eye gaze equipment may have made the task more demanding. But overall, the accuracy levels are very similar to what has been reported in some of the literature studies (Edelstein et al., 2006; Porter and Ten Brinke, 2008; Swerts et al., 2013; Serras Pereira et al., 2014).

The automated movement analysis revealed that there was a positive correlation between the overall amount of movement and the perception of lies, i.e., the more movement the children exhibited during a clip, the higher was the chance that the clip was perceived as a lie. Furthermore, a similar but less strong correlation was found in the body region analysis, which suggests a “gestalt effect” (the whole is more than the sum of its parts) – the more movement the children exhibited in the three different body regions (face, trunk, and legs) in the clips, the more likely it was that it was also perceived as a lie, but less likely when compared with the overall movement correlation. These results contradict partly the argument that people tend to constrain their movements, and show less body motion when lying, as reported by previous studies (DePaulo, 1988; Burgoon, 2005; Duran et al., 2013). However, these previous findings are related with adult’s deceptive behavior, and should be carefully considered when comparing to children’s’ deceptive behavior, since these differences might be related to the age difference. Moreover, this method suggests an interesting difference in non-verbal behavior between the children’s first and second attempt to produce a lie. While the overall amount of movement appears not be distinct from the one in the truthful condition during the first attempt, there does appear to be a difference during the second attempt. Furthermore, when focusing on specific regions of the body, it appears that this behavioral pattern generalizes to different body parts. During the second attempt to produce a lie, there is a significant increase of movement in the head, trunk, and legs that distinguish it from the truthful condition, which does not happen between the truthful and the first lying condition. Additionally, there is more movement happening on the trunk and head when compared with the legs, which seems to indicate that most of the movement happens in the upper part of the body. The non-significant movement differences between the head and the trunk might be explained by this fact, and it might indicate that the head and trunk work as a full unit/block in terms of movement expression. In any case, these findings appear to be in line with earlier finding (Swerts et al., 2013) that a child’s awareness of the fact that it is producing a lie leads to the ironic fact that it becomes harder to hide non-verbal cues to deception: They tend to leak more cues because of the irony effect.

Moreover, the heat map and the focus map visualizations from the movement analysis point toward the same body regions in which the judges, from the first study, thought they based their decision, when deciding whether a clip is truth or a lie. The face (75.62%) was the most often reported region but the feet (33.40%) and legs (30.35%) also seemed to play a significant role. These findings are also supported and corroborated by the eye-tracking study (second study). Although the body tends to leak more movements during a deceptive situation, it seems that the judges mainly focus on the face when deciding if one is being truthful or not. These findings are partly in line with previous research (Bond, 2008).

Lastly, the eye-tracking study revealed that, even when there is non-verbal leakage (movement) happening in different body regions, as illustrated by the heat and focus maps (**Figures 5** and **6**), it seems that judges tended to limit their main focus of attention to only a limited part of the body, namely the face region. Yet, what is not clear is whether the judges chose to ignore (in a more or less conscious way) these non-verbal leaks, or if the movement on those regions is not informative enough for making the decision, or if the judges use their peripheral vision toward those regions, when looking to the face. To further understand these phenomena and to clarify whether the movement on the different regions is informative enough for lie detection, we are currently conducting new perceptive studies where only parts of the body (face, body and feet) are shown to participants.

## Limitations

We would also want to discuss some of the limitations of the current study. The first one is related to the experimenter role. The data were obtained through a paradigm in which a “human” experimenter participated, very much in line with previous studies in this area (see e.g., Talwar and Lee, 2002a,b). Although the experimenter tried to be as consistent and neutral as possible throughout the entire study, she is obviously not acting like a robot that uses a limited and controlled set of interaction strategies. There are several aspects that contribute to this factor: first of all, in the interactive setting, the experimenter is likely to adapt to characteristics and perceived personality of the interacting child. In the present study, there was obviously quite some variability in the way the children behaved, so that it becomes almost unavoidable that these, maybe even unconsciously, have influenced the way the experimenter interacted with those children. For example, think about children that are friendlier and smile more during their interaction, versus children that were very quiet and shy throughout the entire game. These factors may have influenced the way the experimenter behaved. One could consider using a robot or an avatar instead of a human experimenter like in previous studies (e.g., Swerts, 2012; Serras Pereira et al., 2016) as this would allow control over the experimenter role, which might conversely introduce a certain risk that the interaction would become more artificial, and thus leading to data that are not ecologically valid. More work is needed here. Furthermore, we have limited the study to Portuguese children without really controlling for gender, so that it would seem obvious to extent the study to include other factors, such culture and age, into the analyses, to explore whether these have an effect on children’s behavior. Finally, there are also technical limitations. For instance, the eye-tracking study showed that judges tend to focus on the facial area while trying to detect a lie. While this suggests that observers were primarily looking for behavioral cues in that bodily region the method does not allow to exclude the possibility that observers were detecting cues in other bodily areas as well through more peripheral vision. A more sophisticated method that takes such peripheral viewing into account would therefore seem useful. Along the same lines, our frame-differencing method has given us first crude evidence that bodily movement is used as a cue by observers for lie detection. This method could be fine-tuned so that it is able to provide more exact details on patterns in bodily motions that are associated with truthful and deceptive behavior.

## Conclusion

In sum, the present study examined how easily it can be detected whether a child is being truthful or not in a game situation, and it explores the cue validity of body movements for such type of classification. To accomplish this, an original methodology was used, i.e., the combination of perception studies (in which one uses eye-tracking technology) and automated movement analysis. Film fragments from truthful and deceptive children were shown to human judges who were given the task to decide whether the recorded child was being truthful or not. Results showed that, in a set of perception studies, judges were able to correctly distinguish truthful clips from lying clips. Despite the fact that the automated movement analysis for overall and specific body regions did not yield significant results between the experimental conditions, a positive correlation between the amount of movement in a child and the perception of lies was found. This means that the more movement the children exhibited during a clip, the higher the chance that the clip was considered a lie. Finally, the eye-tracking study revealed that judges tend to focus their attention mainly on the face region, even if there is movement happening in different body regions as well.

Finally, contrary to what earlier research has stated, it seems that body movement is a good source for the detection of deception. The frame differencing method used in the current study proved that children tend to show more body movement during a lie-tell. However, a more sophisticated and robust movement analysis is desired for future studies. This type of analysis will allow to further understand and differentiate the type of body movement during a deceptive situation. Also, in order to further understand which are the facial expressions that correlate with children’ lying behavior, a systematic and automated facial expressions analysis is desirable. Being able to identify these facial expressions can be an important step toward efficient lie detection. Furthermore, it would be useful to understand how children’s verbal behavior during deceptive interactions correlates with deception detection. In particular, how disfluencies like pauses or/and acoustic properties, such as pitch and intonation relates to deception. Finally, note that the child participants in our study were Portuguese, whereas the judges were Dutch. In the future, it would be nice to explore whether there are any cross-cultural differences in the expression and detection of deception.

## Author Contributions

MSP, SS, and MS designed the research. MSP performed the research. MSP, MS, RC, and EP analyzed the data. MSP, SS, and MS wrote the article.

## Conflict of Interest Statement

The authors declare that the research was conducted in the absence of any commercial or financial relationships that could be construed as a potential conflict of interest.
