# Supplementary material for: Comparing a Perceptual and an Automated Vision-Based Method for Lie Detection in Younger Children
Source: Front Psychol. 2016 Dec 12;7:1936. doi: 10.3389/fpsyg.2016.01936 (PMC5149550; doi:10.3389/fpsyg.2016.01936)
Supplement: Supplementary file 1 [file Script.PDF]

```
% FrameDifferencing.m
% script for performing frame differencing on videos
% written by Eric Postma (eric.postma@gmail.com/www.ericpostma.nl)
% for Matlab versions 2013 and up

% The result of the script is a matrix (AvgMatrix) with dimensions
corresponding to the
% dimensions of the video frames. Each element of the matrix has a
value corresponding to
% the average change at the corresponding the pixel location in the
video.

indirname = 'clips'; % directory containing the video files
outdirname = 'output directory';

threshold = 25;

videos = dir([indirname '/*.mov']);

for v=1:length(videos)

    if ~exist([outdirname '/' videos(v).name(1:end-4)
'.mat'],'file')

        % Things may go wrong here if you don't have the proper
codec
        VidObject = VideoReader([indirname '/' videos(v).name]);

        PreviousFrame = read(VidObject,1);

        % We assume color video and collapse rgb channels
        PreviousFrame = mean(double(PreviousFrame), 3);
        % PreviousFrame = zscore(PreviousFrame);

        rw = size(PreviousFrame,1);
        cw = size(PreviousFrame,2);

        % Allocation of matrix for staring FD results
        % requires sufficient RAM memory
        MovMatrix = zeros(rw,cw);
        MoveVector = zeros(VidObject.NumberOfFrames,1);
        AvgMatrix = zeros(rw,cw);

        for frame=2:VidObject.NumberOfFrames-1;
            disp(['Processing ' videos(v).name ' (' int2str(v) '/'
int2str(length(videos)) ') Frame ' int2str(frame) ' of '
int2str(VidObject.NumberOfFrames)]), drawnow;

            CurrentFrame = read(VidObject,frame);
            CurrentFrame = mean(double(CurrentFrame), 3);

            % Compute the pixelwise differences between subsequent
frames
            DiffFrame = abs(PreviousFrame-CurrentFrame);
```

```
            % Suppress noise-induced changes below threhold
            DiffFrame(DiffFrame(:)<threshold)=0;

            % Add the differences to AvgMatrix
            AvgMatrix = AvgMatrix+DiffFrame;

            % The current frame will becoe the previous frame
            PreviousFrame = CurrentFrame;
        end;

        % Normalize AvgFrame
        AvgFrame = AvgFrame/(VidObject.NumberOfFrames-1);

        % Save results
        save([outdirname '/' videos(v).name(1:end-4) '.mat'],
'MoveVector','AvgMatrix');
    end;
end;
```
